# Research Advances in Glanimal Models of Glaucoma: Exploring Multidimensional Mechanisms and Novel Therapeutic Strategies

**DOI:** 10.3390/pharmaceutics18020152

**Published:** 2026-01-25

**Authors:** Jinshen Liu, Hui Zhang, Jiaqi Chen, Jiamin Zhou, Yujia Yu, Feng Cheng, Jie Bao, Chunhan Feng, Xiangqu Yu, Zhao Xia, Rao Ding, Zhonghui Li, Xiang Li

**Affiliations:** 1 Eye School, Chengdu University of Traditional Chinese Medicine, Chengdu 610032, China; liujinshen@stu.cdutcm.edu.cn (J.L.);; 2 Ophthalmology Department, Hospital of Chengdu University of Traditional Chinese Medicine, Chengdu 610075, China

**Keywords:** glaucoma, animal models, retinal ganglion cells, intraocular pressure, neuroprotection, immune inflammation, ischemia–reperfusion, pharmacotherapy

## Abstract

**Objective**: Glaucoma is a complex optic neuropathy characterized by the progressive loss of retinal ganglion cells (RGCs). Animal models are crucial tools for deciphering its multidimensional pathogenesis and evaluating novel therapeutic strategies. This review aims to systematically summarize the establishment methods, application advances, and future development trends of various glanimal models. **Methods**: The literature for this review was identified through systematic searches of electronic databases, including PubMed, Web of Science Core Collection, and Google Scholar. The search strategy utilized a combination of keywords and their variants: “glaucoma”, “animal models”, “retinal ganglion cells”, “intraocular pressure”, “neuroprotection”, “immune inflammation”, “fibrosis”, and “filtration surgery”. The search focused on articles published between 2015 and 2025 to cover the major advances of the last decade. The scope encompassed original research articles, reviews, and meta-analyses. **Results**: Diverse glanimal models successfully replicate different facets of glaucoma, elucidating multidimensional pathogenesis involving mechanical stress, immune inflammation, excitotoxicity, oxidative stress, and fibrosis. These models have played an indispensable role in screening neuroprotective agents, evaluating anti-fibrotic strategies, and validating the application of advanced imaging and functional assessment technologies. Current research is evolving towards model standardization, multi-factor simulation, and the integration of novel drug delivery systems and immunomodulatory strategies. **Conclusions**: The diversification of glanimal models provides a powerful platform for in-depth investigation of disease mechanisms and the development of innovative therapies. Future research should focus on establishing standardized models that better mimic the clinical pathological state and deeply integrating multimodal assessment technologies with targeted therapies. This will facilitate the translation of basic research into clinical applications, ultimately achieving personalized precision medicine for glaucoma.

## 1. Introduction

As a leading cause of irreversible blindness worldwide, glaucoma is pathologically characterized by persistent degenerative damage to retinal ganglion cells (RGCs) and optic nerve fibers, severely impacting patients’ visual function [1]. Epidemiologically, glaucoma exhibits notable variations in prevalence across racial and age groups [2]. Primary open-angle glaucoma (POAG) is more prevalent in individuals of African descent, while angle-closure glaucoma is more common in East Asian populations [3]. Age is a significant risk factor, with incidence increasing markedly after 40 years and rising sharply in those over 60 [4]. Although elevated intraocular pressure (IOP) is considered the primary risk factor for glaucoma onset, clinical and experimental studies indicate that normal tension glaucoma (NTG) and non-IOP-related mechanisms also play significant roles in the disease process [5,6]. Optic nerve damage in NTG patients often occurs within the normal IOP range, suggesting that factors beyond IOP, such as hemodynamic abnormalities, immune responses, and genetic factors, contribute to the pathology [7,8].

**Figure 1:**
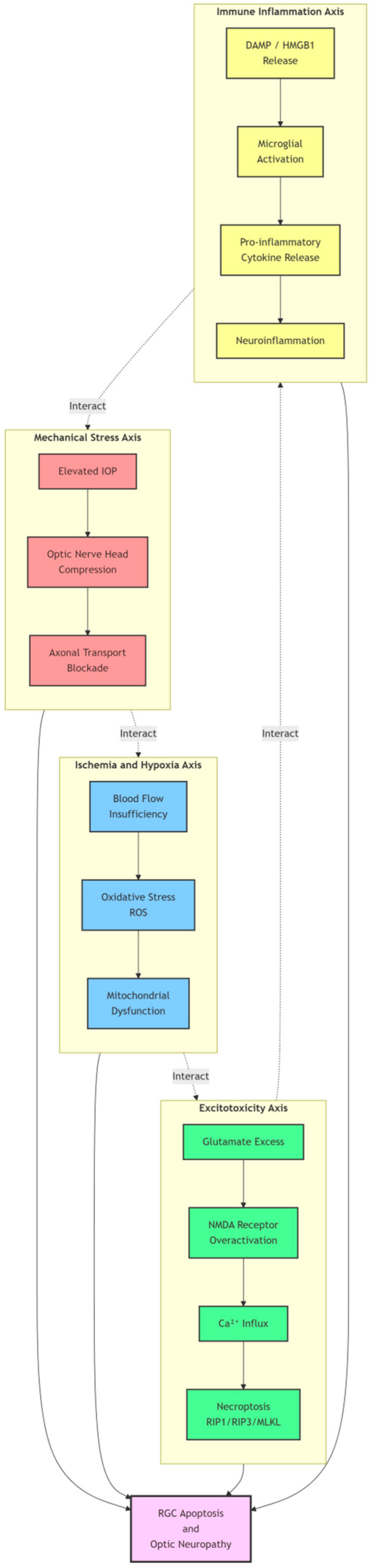
Schematic diagram of the multidimensional pathogenesis of glaucoma.

As shown in Figure 1, the pathogenesis of glaucoma involves multidimensional pathophysiological processes. Beyond the traditional mechanical pressure theory, mechanisms such as ischemia/hypoxia, excitotoxicity, and immune inflammation collectively form a complex pathological network. Understanding this multidimensional mechanism is crucial for developing more effective therapeutic strategies. Figure 1 clearly illustrates the interactions among these four core pathological axes, providing an intuitive framework for understanding the complex pathogenesis of glaucoma.

Animal models serve as vital platforms for studying the complex pathogenesis of glaucoma and developing therapeutic strategies, and have been widely used and developed in recent years. In this review, we use the term ‘Glanimal models’ (from glaucoma and animal) to specifically denote animal models developed and used for glaucoma research. Notably, several animal models have demonstrated high translational value and success in elucidating disease mechanisms and testing therapies [9]. For instance, the DBA/2J mouse model, a widely adopted hereditary model, has been instrumental in uncovering the role of immune dysregulation and complement activation in glaucomatous neurodegeneration, directly contributing to the development of complement-targeted therapeutic strategies [10]. Another prominent example is the glucocorticoid (GC)-induced glaucoma model. Weekly periocular injections of dexamethasone-21-acetate (Dex-Ac) in mice reliably induce ocular hypertension, retinal ganglion cell loss, and optic nerve degeneration. This model recapitulates key features of POAG, including reduced aqueous outflow due to trabecular meshwork dysfunction with extracellular matrix deposition, making it an ideal system for studying both upstream pathophysiology in the anterior segment and downstream neurodegenerative events, as well as for screening novel therapeutic interventions [11]. Through animal models, researchers can simulate different types of glaucomatous pathological states, thereby enabling in-depth exploration of the multidimensional mechanisms underlying optic nerve degeneration [12,13]. Existing glanimal models encompass various types, including genetic mutation models, pharmacologically induced models, mechanically induced IOP elevation models, immune-mediated models, and ischemia–reperfusion models, each with specific pathological manifestations and research value. For instance, genetic models like the DBA/2J mouse and LTBP2 mutant cat model reflect genetically related glaucomatous processes; pharmacologically induced models mimic IOP elevation through drug intervention; and immune-mediated models are used to study the role of the immune system in optic nerve injury [6,13,14].

Furthermore, neuroprotective strategies have become a hot topic in glaucoma treatment research in recent years. Stem cell therapy, due to its multipotent differentiation potential and ability to secrete neurotrophic factors, has shown significant efficacy in animal experiments in regulating IOP, protecting RGC survival, and maintaining the structural integrity of the nerve fiber layer [1]. Additionally, novel drug developments are continuously emerging. For example, HL3501, a selective A3 adenosine receptor antagonist, demonstrates good IOP-lowering effects and anti-fibrotic activity, providing a new candidate drug for clinical treatment [15]. Laser therapies, such as selective laser trabeculoplasty (SLT), have also shown some IOP-lowering effects in NTG patients, although their long-term efficacy requires further validation [16].

Notably, blood pressure fluctuations, diurnal IOP variations, and local hemodynamic abnormalities in the optic nerve are considered important non-IOP factors in NTG pathogenesis. Multiple studies using dynamic monitoring have found that NTG patients often exhibit dysregulated blood pressure, abnormal nocturnal blood pressure dips, and elevated morning blood pressure peaks, which are closely related to the progression of optic nerve injury [17,18]. Concurrently, 24 h IOP fluctuations and sleeping posture are also associated with NTG pathogenesis. Continuous monitoring using contact lens sensors revealed that the amplitude of IOP fluctuations and the duration of certain sleeping positions were significantly greater in NTG patients compared to healthy controls [8,19]. These findings emphasize the importance of assessing and managing non-IOP-related factors to improve the overall treatment strategy for glaucoma.

In summary, the pathogenesis of glaucoma is complex, involving not only IOP-related mechanical compression and ischemia but also multiple factors such as immune inflammation, lack of neuroprotection, and hemodynamic abnormalities. Animal models, as key tools for studying these multidimensional mechanisms, not only enhance the understanding of the disease essence but also provide an important platform for screening and evaluating novel therapeutic approaches. Future research needs to further integrate multidisciplinary methods, optimize animal model design, and promote the translation from basic research to clinical applications, ultimately bringing more effective prevention and treatment strategies to glaucoma patients. The aim of this review is to systematically summarize the establishment methods, pathophysiological insights gained, and therapeutic applications of various animal models of glaucoma, while discussing future directions and challenges in the field.

## 2. Literature Search Strategy and Selection Criteria

This review was conducted based on a systematic search of the literature. Electronic databases, including PubMed, Web of Science Core Collection, and Google Scholar, were searched for relevant articles published between January 2015 and March 2025. The search strategy utilized a combination of the following keywords and their variants: “glaucoma”, “animal models”, “retinal ganglion cells”, “intraocular pressure”, “neuroprotection”, “immune inflammation”, “fibrosis”, and “filtration surgery”. The search was limited to articles published in English. Inclusion criteria were: (1) original research articles, reviews, or meta-analyses focusing on the establishment, validation, or application of animal models for glaucoma; (2) studies investigating the pathophysiology of glaucoma, including mechanisms of RGC injury, neuroprotection, inflammation, or fibrosis in animal models; and (3) research employing advanced imaging or functional assessment techniques in glaucoma animal models. Exclusion criteria were: (1) studies not involving animal models; (2) case reports, editorials, or conference abstracts without full text; and (3) studies published before 2015 or in languages other than English.

## 3. Classification and Establishment Methods of Glanimal Models

Animal models are indispensable for replicating the complex and multifactorial pathology of glaucoma. They allow researchers to isolate specific etiological factors, study disease progression in a controlled setting, and test potential interventions. The choice of model depends critically on the research question, whether it pertains to genetics, intraocular pressure dynamics, immune mechanisms, or ischemic injury. This section categorizes and details the major types of animal models, their establishment methods, and their respective applications and limitations.

Table 1 systematically compares the characteristics of four major types of glanimal models. The selection of these models is based on a comprehensive review of the literature from 2015 to 2025, focusing on their widespread adoption, ability to replicate specific facets of glaucoma pathology (e.g., chronic IOP elevation, immune involvement, ischemia, genetic predisposition), and their demonstrated utility in mechanistic studies and therapeutic screening. Genetic models spontaneously simulate human disease progression but are costly; IOP elevation models are operationally simple but may introduce non-specific injury; immune-mediated models are suitable for studying non-IOP mechanisms but have relatively singular pathology; ischemia/axonal injury models are suitable for neuroprotection research but differ from chronic progression. Researchers should select appropriate models based on specific scientific questions.

**Figure 2:**
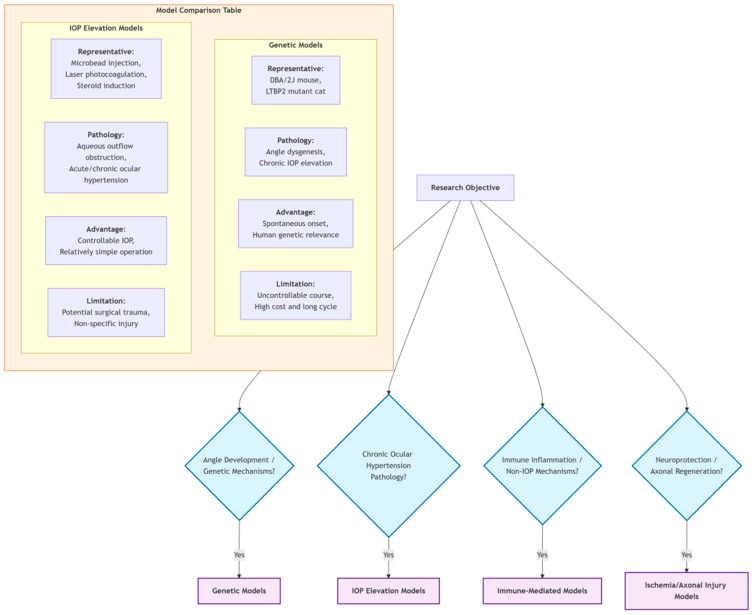
Overview of Establishment Methods and Applications of Major Glanimal Models.

Figure 2 systematically outlines four major types of glanimal models. The table below compares the representative methods, simulated pathology, advantages, and limitations of each model type. The flowchart above provides a model selection pathway: choose genetic models for studying angle development and genetic mechanisms; choose IOP elevation models for studying chronic ocular hypertension pathology; choose immune-mediated models for studying immune inflammation and non-IOP mechanisms; choose ischemia/axonal injury models for studying neuroprotection and axonal regeneration.

### 3.1. Hereditary Glaucoma Models

Hereditary glanimal models play a significant role in simulating the pathogenesis of congenital and pigmentary glaucoma. Among these, the DBA/2J mouse is one of the most commonly used natural genetic models. This mouse model carries multiple gene mutations leading to typical manifestations of pigmentary glaucoma, such as abnormal angle structure and elevated IOP. Studies show that concentrations of tryptophan (TRP) and its metabolites like kynurenic acid (KYNA) and kynurenine (KYN) are significantly reduced in the retina and serum of DBA/2J mice, suggesting abnormalities in the tryptophan metabolic pathway may participate in the neurodegenerative pathology of hereditary glaucoma [20]. Furthermore, the DBA/2J model exhibits obstruction of the aqueous outflow pathway due to angle dysgenesis, causing IOP elevation, which resembles the pathological mechanism of human pigmentary glaucoma.

Another important hereditary model is the LTBP2 gene mutant cat, which simulates congenital glaucoma caused by angle dysgenesis. Mutation in the LTBP2 gene affects the development of intraocular structures, particularly the normal formation of the angle and iris, leading to impaired aqueous outflow, elevated IOP, and subsequent glaucoma. Related studies also found that LTBP2 is closely associated with the transforming growth factor-beta (TGF-β) signaling pathway, which plays a key role in regulating IOP and angle cell function; its aberrant activation may exacerbate the condition [21].

The mechanisms by which gene mutations affect angle dysgenesis and IOP regulation primarily involve the proliferation, differentiation of angle tissue cells, and the regulation of the extracellular matrix. Abnormal gene expression leads to cellular dysfunction, disrupting the integrity of the angle structure, thereby affecting normal aqueous outflow, causing IOP elevation and optic nerve injury. Additionally, gene mutations may alter cellular metabolic states, enhance oxidative stress responses, promote angle cell apoptosis and neuronal degeneration, further aggravating glaucoma progression [22,23].

In recent years, gene editing technologies, particularly the CRISPR/Cas system, have shown great potential in constructing precise hereditary glanimal models. This technology enables the simulation of glaucoma-related gene mutations through targeted modification of specific pathogenic genes, allowing for more accurate replication of the human genetic background and pathological features of glaucoma. Furthermore, CRISPR technology is also applied in the development of gene therapy strategies, attempting to correct pathogenic genes or modulate related signaling pathways to restore normal angle function and IOP regulation, holding broad prospects for clinical translation [24,25]. In the future, combining CRISPR technology with induced pluripotent stem cell (iPSC) technology will enable more personalized and precise disease modeling, facilitating in-depth revelation of the molecular mechanisms of hereditary glaucoma and screening effective targeted drugs, providing new ideas and methods for clinical treatment.

### 3.2. Intraocular Pressure Elevation Induction Models

IOP is a major risk factor for glaucoma onset. Animal models simulating IOP elevation are crucial for studying glaucoma pathogenesis and treatment strategies. Commonly used methods for inducing IOP elevation include microbead injection, laser photocoagulation, and pharmacological induction.

The microbead injection method involves injecting micron-sized beads into the anterior chamber to obstruct the trabecular meshwork outflow pathway, impairing aqueous humor drainage and consequently elevating IOP. This method is widely used in various rodents like rats and mice. For example, one study successfully elevated IOP from 9.3 ± 0.1 mmHg to 20.8 ± 1.6 mmHg for 8 weeks by weekly intracameral microbead injection in rats, inducing degenerative changes in RGCs and the optic nerve [26]. The microbead model effectively simulates clinical chronic IOP elevation, and retinal proteomic analysis revealed that IOP elevation triggers neurodegenerative mechanisms including decreased glutathione metabolism, mitochondrial dysfunction, and enhanced oxidative stress.

Laser photocoagulation involves laser application to the angle tissues or episcleral veins in small animals, obstructing aqueous outflow to cause IOP elevation. This method can create functional aqueous drainage obstruction, leading to gradual IOP increase and corresponding retinal/optic nerve damage. A study in rodents proposed a progressive IOP elevation model using a modified circumlimbal suture technique, resulting in slow and sustained IOP elevation without acute spikes, thus more closely mimicking the clinical chronic glaucoma process [27]. This model significantly induced retinal nerve fiber layer (RNFL) thinning, optic nerve head remodeling, and RGC loss, accompanied by inflammation in the retina and optic nerve, suggesting the IOP elevation pattern significantly impacts pathological progression.

Pharmacologically induced IOP elevation models are represented by steroid hormone (e.g., dexamethasone) injection or sustained-release microsphere delivery. The steroid-induced glaucoma model mimics clinical steroid-induced glaucoma features, including IOP elevation, RGC apoptosis, and optic nerve degeneration. One study compared two steroid induction models over a long period (24 weeks), finding more significant IOP elevation in males and differences in retinal layer thickness changes and electrophysiological parameters between models (e.g., dexamethasone microspheres vs. dexamethasone-fibronectin complex microspheres) [28]. Furthermore, local or systemic medications (e.g., pioglitazone, baicalin) showed potential in protecting RGCs, reducing oxidative stress, and modulating signaling pathways in such models [29,30].

Different induction methods vary in the persistence and magnitude of IOP elevation. Microbead models typically produce moderate, sustained IOP elevation, while laser models can achieve slow or rapid elevation depending on parameters. Steroid models more reflect drug-induced IOP elevation with greater fluctuation. Additionally, different models exhibit variations in retinal and optic nerve damage manifestations, such as the extent of optic nerve head remodeling, RNFL thinning, and RGC loss [31,32].

IOP fluctuation is considered an important factor influencing glaucoma progression. Studies show that short-term IOP fluctuations and peaks are associated with retinal ischemia and neural damage, potentially exacerbating RGC apoptosis and retinal dysfunction [33]. In some IOP elevation models, IOP fluctuations promote retinal ischemia and inflammatory responses, thereby affecting disease severity and progression rate. Moreover, uneven distribution of IOP between the anterior chamber and vitreous cavity may exert different mechanical stresses on the optic nerve head, influencing the pattern of neural damage [34].

In summary, IOP elevation-induced animal models encompass various methods, each with distinct characteristics, simulating different stages and types of glaucoma pathology. Systematic study of the persistence, magnitude of IOP elevation, and differences in retinal/optic nerve damage across models facilitates a deeper understanding of glaucoma pathogenesis, exploration of the relationship between IOP fluctuations and retinal ischemia, and provides experimental basis for developing novel therapeutic strategies.

### 3.3. Immune-Mediated and Excitotoxicity Models

Immune-mediated and excitotoxicity models are important non-IOP-only models in glaucoma research. They simulate abnormal immune system responses and neuronal excitotoxic processes, revealing multidimensional mechanisms of RGC apoptosis.

Firstly, experimental autoimmune glaucoma models often involve immunization with retinal antigens to induce injury. For instance, systemic immunization with optic nerve antigen (ONA) can lead to RGC loss and retinal function decline. Studies show that after immune activation, early complement system activation, particularly deposition of the membrane attack complex, promotes optic nerve injury and nerve fiber degeneration [35]. Additionally, upregulation of heat shock protein family members and immune-related genes (e.g., C1qa, Il18, Nfkb1) in autoimmune models suggests a key role of immune inflammation in glaucoma pathogenesis [36]. In these models, activation of immune cells like microglia and macrophages accompanies increased pro-inflammatory cytokines (e.g., TNF-α, IL-1β), promoting RGC apoptosis [37]. Furthermore, immunodeficient mice (e.g., Rag1-/-) show significant protection against immune-mediated RGC loss due to the lack of T and B cells, further confirming the role of adaptive immunity in glaucoma progression [38]. Retinal and optic nerve glial cells (astrocytes and Müller cells) exhibit gliosis during immune activation, releasing inflammatory factors and participating in RGC neurotoxicity [37,39].

Secondly, the N-methyl-D-aspartate (NMDA) injection model is a classic method for excitotoxicity research. NMDA, as a glutamate receptor agonist, causes rapid RGC apoptosis, simulating excitotoxic damage to the optic nerve in glaucoma. In vivo and in vitro studies confirm that NMDA-induced excitotoxicity leads to intracellular calcium overload, activating the RIP1/RIP3/MLKL pathway-mediated necroptosis, accompanied by NLRP3 inflammasome activation, exacerbating inflammation and cell death [40]. Using RIP1 and RIP3 inhibitors (e.g., GSK872 and Necrostatin-1) effectively protects RGCs and reduces inflammation, suggesting excitotoxicity-mediated necroptosis is a potential therapeutic target [40]. Additionally, PF4 (a platelet factor) exhibits neuroprotective effects in the NMDA model, inhibiting microglial inflammatory responses and promoting the CaMKII/CREB/BDNF pathway, thereby mitigating RGC apoptosis [41]. Other studies found that FABP5 regulates the ROS-NLRP3 inflammasome pathway, participating in NMDA-induced retinal excitotoxic injury, and FABP5 inhibitors reduce RGC loss [42]. Furthermore, SB202190, an inhibitor of the p38 MAPK pathway, can suppress the ferroptosis process, alleviating NMDA-induced RGC damage, revealing the link between excitotoxicity and diverse cell death mechanisms [43]. Other drugs like Ripa-56 and melatonin also show potential in reducing excitotoxicity and protecting RGCs [44,45].

Finally, the role of immune inflammation in glaucoma pathogenesis is increasingly recognized. Microglia, astrocytes, and Müller cells activate in the retina and optic nerve head, releasing various inflammatory cytokines (e.g., TNF-α, IL-1β) and chemokines, participating in inflammatory amplification and neurodegenerative injury [37,46]. Spatiotemporal dynamics of retinal immune cells indicate that immune cell-mediated inflammatory responses play important roles in both early and late stages of RGC injury [47]. Complement system dysregulation is also considered a key factor in chronic inflammation and neurodegeneration; complement inhibitors show protective effects on optic nerve structure in autoimmune glaucoma models [35,48]. Moreover, using vitreous optical coherence tomography (OCT) to monitor immune cell morphology and dynamics provides new avenues for non-invasive monitoring of immune inflammation [49,50].

In summary, immune-mediated and excitotoxicity models, by simulating abnormal immune activation and excessive glutamate receptor stimulation, reveal the complex neuroinflammation and neurotoxicity mechanisms in glaucoma. Immune cell activation is closely related to cytokine changes, and excitotoxicity-induced RGC death involves multiple cell death pathways. Therapeutic strategies targeting these mechanisms, such as inhibiting complement activation, modulating glial cell responses, and blocking necroptosis and ferroptosis pathways, show promising application prospects, providing an important foundation for multidimensional mechanism research and novel therapeutic strategy exploration in glaucoma [13,40,41].

### 3.4. Ischemia–Reperfusion and Optic Nerve Crush Models

The ischemia–reperfusion (I/R) model involves transiently elevating IOP to induce retinal ischemia, followed by reperfusion, thereby simulating retinal and optic nerve I/R injury. This model can rapidly induce damage to the retina and optic nerve, leading to RGC death and neural dysfunction. The I/R model is particularly important in glaucoma research as it simulates not only mechanical damage from high IOP but also reveals multiple mechanisms like ischemia, oxidative stress, and inflammation in neurodegeneration. For example, studies show that after I/R injury, the retinal N-methyl-D-aspartate receptor NR2B subunit and its interaction with postsynaptic density protein PSD95 significantly increase, exacerbating excitotoxicity and RGC apoptosis. Inhibiting the NR2B-PSD95 interaction effectively protects RGCs and reduces retinal damage [51]. Furthermore, the local retinal renin–angiotensin–aldosterone system (RAAS) is activated after I/R injury, inducing oxidative stress and inflammation; using AT1 receptor antagonists can alleviate these pathological changes, suggesting a key role of RAAS in I/R injury [52]. Therapeutically, small molecule peptides like peptain-1 can inhibit inflammation-mediated apoptosis and reduce retinal capillary degeneration, possessing potential neuroprotective value [53]. The I/R model is also widely used to evaluate the neuroprotective effects of drugs, such as NCX 470, which protects retinal cell function by improving retinal hemodynamics and reducing oxidative stress [54]. In summary, the I/R model effectively simulates multidimensional injury mechanisms in glaucoma, including retinal ischemia, oxidative stress, and inflammation, providing an ideal platform for evaluating neuroprotective drugs.

The optic nerve crush (ONC) model involves mechanically compressing the optic nerve to induce axonal injury, simulating the pathological process of optic nerve damage in normal tension glaucoma (NTG). This model is characterized by direct acute axonal injury, leading to RGC axonal disruption and apoptosis, making it suitable for studying neurodegenerative mechanisms and regenerative potential after axonal injury. Compared to the I/R model, ONC focuses more on the axonal level of injury and subsequent cell death processes. Studies show that in the ONC model, activated inflammatory cells, particularly microglia, have complex effects on RGC survival; however, experimental evidence indicates that even complete depletion of microglia does not significantly alter RGC mortality, suggesting microglia may play more of a bystander role in this model [55]. Additionally, in neuroprotection studies based on this model, combined use of cytokines like CNTF and GDNF significantly delays RGC apoptosis and promotes cell survival [56]. Gene therapy strategies, such as BCLXL overexpression, also show potential in protecting RGC somas and their axons in the ONC model [57]. Furthermore, intervention targeting the CCL5/CCR5 signaling pathway inhibits ONC-induced RGC apoptosis, indicating inflammatory signaling pathways are an important component of the neurodegenerative mechanism in this model [58]. The ONC model has also been used to assess the impact of hormonal changes on optic nerve injury, such as surgical menopause exacerbating visual function decline after ONC [59]. Therefore, the ONC model provides an important experimental basis for exploring axonal injury mechanisms and neuroprotective strategies in non-high-pressure glaucoma.

I/R and ONC models each have advantages in glanimal model research. The I/R model effectively simulates retinal ischemia and related inflammation/oxidative stress processes due to high IOP, making it suitable for studying multidimensional glaucoma pathology and screening anti-ischemic and antioxidant drugs. The ONC model more directly simulates axonal injury, suitable for studying neurodegenerative mechanisms and neuroprotection/regeneration strategies. Both are widely used to evaluate the efficacy of neuroprotective drugs. For example, the anti-inflammatory drug Magnolol achieves neuroprotection in the I/R model by activating PPARγ and inhibiting NF-κB-dependent inflammation [60]; the β-lactam antibiotic ceftriaxone exhibits neuroprotective effects in the I/R model by promoting GLT-1 expression and alleviating excitotoxicity [61]. Moreover, the natural compound resveratrol demonstrates significant protective effects in various retinal disease models, including I/R, through multi-target antioxidant, anti-inflammatory, and neuroprotective mechanisms [62]. Research based on these models continuously enriches the understanding of glaucoma pathogenesis and promotes the development of novel therapeutic strategies. In conclusion, I/R and ONC models provide powerful experimental platforms for neuroprotective drug research in glaucoma, facilitating disease mechanism analysis and therapeutic innovation.

## 4. Research on Neuroprotective Mechanisms in Glanimal Models

Preventing or delaying the death of retinal ganglion cells is the central goal of glaucoma therapy beyond IOP control. Animal models have been pivotal in deciphering the cascade of events leading to RGC apoptosis, including oxidative stress, excitotoxicity, and neuroinflammation. This section explores these injury mechanisms and reviews how animal models serve as crucial platforms for screening and evaluating novel neuroprotective drugs and immunomodulatory strategies.

### 4.1. Exploration of Retinal Ganglion Cell Injury Mechanisms

RGC injury is the core event in glaucoma pathogenesis. Their death mechanisms involve various complex cellular and molecular pathways, primarily including oxidative stress, excitotoxicity, and inflammatory responses. Firstly, oxidative stress plays a significant role in RGC injury in glaucoma. Excessive reactive oxygen species (ROS) production leads to lipid peroxidation, protein damage, and DNA breakage, triggering apoptosis and cellular dysfunction. For instance, NADPH oxidase (NOX) is a significant source of ROS, and its increased expression in glaucoma models exacerbates oxidative stress, inducing RGC death [63]. Additionally, metal ions like iron and zinc regulate mitochondrial function and metabolic homeostasis; their imbalance can worsen mitochondrial damage and energy metabolism disorders in RGCs, accelerating optic neurodegeneration [64]. On the other hand, excitotoxicity also contributes to RGC death. Excessive glutamate overactivates NMDA receptors, leading to disrupted Ca2+ influx and abnormal intracellular signaling, triggering apoptosis. Blocking NMDA receptors or modulating Ca2+ channels (e.g., L-type and T-type voltage-gated calcium channels) has been shown to alleviate RGC injury [65,66]. Inflammatory response is another key factor. Activation of microglia and Müller cells leads to the release of pro-inflammatory cytokines (e.g., TNF-α, IL-1β), which exacerbate RGC damage by promoting neuroinflammation. TNF-α induces Müller cells to secrete the chemokine CCL2, promoting microglial activation and migration, thereby aggravating retinal neuroinflammation [67]. Furthermore, S100A9 protein, as a pro-inflammatory factor, is upregulated in glaucoma, activating the TLR4 signaling pathway and promoting inflammatory responses in microglia and astrocytes, further driving RGC apoptosis [68]. Receptor-interacting protein kinase 1 (RIP1)-mediated necroptosis has also been confirmed to participate in inflammatory RGC death, and its inhibition helps protect RGCs and reduce retinal inflammation [69].

Integrating proteomic and gene expression data, several key regulatory factors have been identified as important nodes controlling RGC death. Among them, high mobility group box 1 (HMGB1), as a damage-associated molecular pattern (DAMP), is released upon retinal injury, inducing inflammation; transforming growth factor-beta 2 (TGF-β2) is elevated in the aqueous humor of glaucoma patients, participating in fibrosis and retinal environment remodeling [70]. Genomic studies show significant changes in the gene expression profiles of RGCs in glaucoma models, involving pathways related to oxidative stress, apoptosis, immune response, and cellular metabolism [71]. Additionally, transcription factors like E2F1 promote a neuroprotective phenotype in Müller cells by regulating secreted phosphoprotein 1 (SPP1) expression, indirectly protecting RGCs [70]. Regulatory proteins like Zfp667 can inhibit the expression of apoptosis-related genes, enhancing RGC survival capacity [72]. Concurrently, the BMP4-GPX4 signaling pathway protects RGCs and promotes retinal stem cell differentiation by alleviating ferroptosis-related oxidative damage [73].

In summary, RGC injury in glaucoma results from the combined action of multiple mechanisms and factors. Oxidative stress, excitotoxicity, and inflammatory responses intertwine, leading to intracellular metabolic imbalance and cell death. Key factors like HMGB1 and TGF-β2 act as signaling hubs, regulating inflammation and apoptosis pathways. Future comprehensive regulation targeting these molecules and pathways, combined with antioxidant, anti-inflammatory, and neuroprotective strategies, holds promise for providing new targets and methods for glaucoma treatment [67,68,70,74].

### 4.2. Screening and Evaluation of Neuroprotective Drugs

Neuroprotective drugs play a vital role in glaucoma treatment, especially in preventing and treating RGC injury and death. In recent years, with advances in molecular biology and drug screening technologies, several candidate neuroprotective drugs, such as Peptains, Oxymatrine, and HL3501, have been validated in animal models. These drugs not only show positive effects in improving RGC survival rates but also promote the recovery of retinal function and maintain structural integrity.

**Figure 3:**
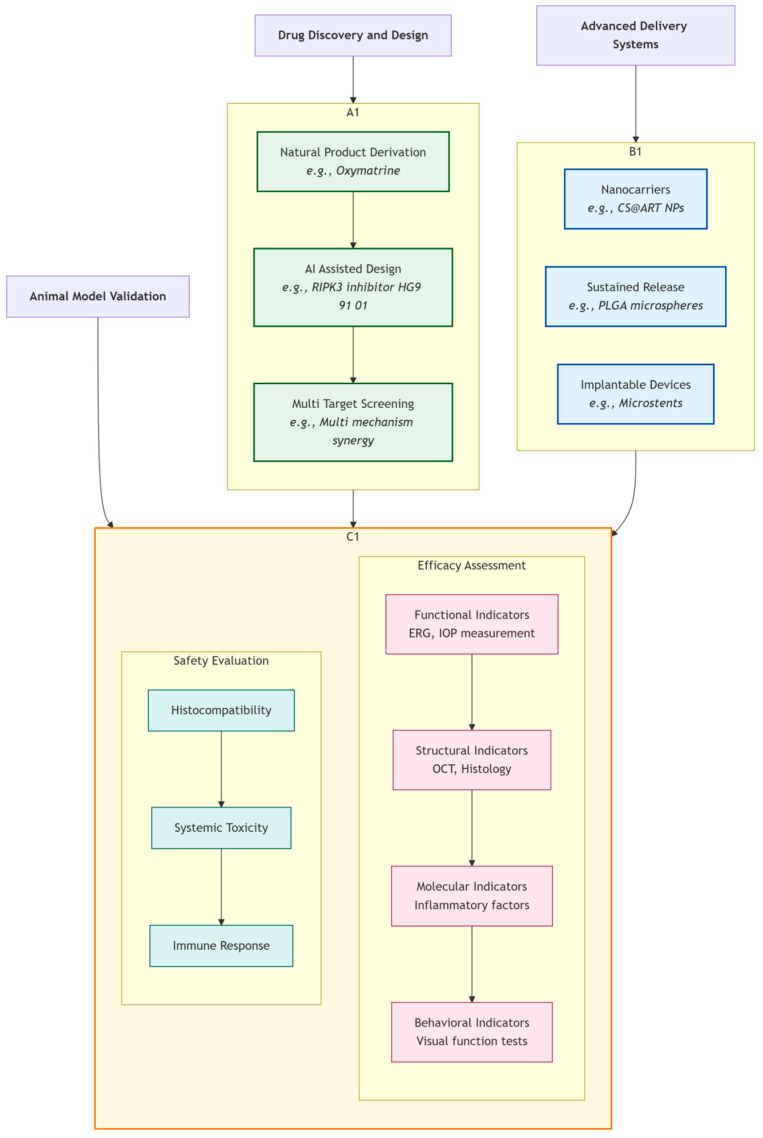
Strategy Chart for the Development of Neuroprotective and Anti-fibrotic Drugs in Glaucoma.

Figure 3 illustrates the complete development chain from drug discovery to preclinical validation. The Drug Discovery and Design stage includes natural product derivation, AI-assisted design, and multi-target screening; the Advanced Delivery Systems stage covers nanocarriers, sustained-release systems, and implantable devices; the Animal Model Validation stage includes efficacy assessment (functional, structural, molecular, and behavioral indicators) and safety evaluation (histocompatibility, systemic toxicity, and immune response).

Peptains are a class of emerging neuroprotective peptides that modulate intracellular signaling pathways like CREB (cAMP response element-binding protein) and SYN1 (Synapsin I), enhancing neuronal survival and functional recovery. In a silicone oil-induced ocular hypertension rat model, Peptain administration significantly alleviated the reduction in retinal layer thickness and increased RGC survival rate, while electrophysiological detection showed it could restore functional retinal indicators, such as ERG waveform parameters [75]. This indicates that Peptains can effectively mitigate neural damage induced by IOP elevation in glaucoma models.

Oxymatrine is a plant-derived natural alkaloid with anti-inflammatory and antioxidant properties. Its derivatives protect neurons from damage by reducing the release of inflammatory factors like IL-1β and TNF-α, thereby inhibiting neuroinflammation. In an Alzheimer’s disease model, Oxymatrine derivatives exhibited strong neuroprotective effects [76]. Although this study focused on neurodegenerative disease, its mechanisms share commonalities with the inflammatory and apoptotic processes in glaucoma, providing new ideas for developing neuroprotective drugs for glaucoma.

HL3501 is a novel drug whose specific research has not been widely reported, but drugs with similar multi-target actions used in animal models suggest that combining multiple neuroprotective mechanisms is more helpful in delaying RGC injury and retinal structural degeneration. For example, multi-drug combination sustained-release systems, particularly those using PLGA (poly(lactic-co-glycolic acid)) microspheres as carriers, enable simultaneous release of multiple neuroprotective drugs, prolonging intraocular drug action time and improving therapeutic efficacy. In one study, PLGA microspheres were used to co-encapsulate dexamethasone (an anti-inflammatory drug), ursodeoxycholic acid (an anti-apoptotic drug), and the neurotrophic factor GDNF. Results showed that this multi-component formulation significantly improved RGC function, maintained retinal nerve layer thickness, and confirmed retinal functional recovery via ERG in a chronic glaucoma model [77]. This combined sustained-release system not only optimized drug release kinetics but also reduced the frequency of multiple administrations, improving patient compliance, and holds broad clinical application prospects.

Furthermore, artificial intelligence (AI)-assisted drug screening is a cutting-edge direction in current neuroprotective drug development. The RIPK3 inhibitor HG9-91-01, screened through AI technology, demonstrated significant neuroprotective effects in an acute IOP elevation glaucoma mouse model, effectively inhibiting various cell death pathways like apoptosis, inflammation, and necrosis in the retina [78]. This provides a new paradigm for the precise design of multi-target neuroprotective drugs.

In summary, the screening and evaluation of neuroprotective drugs rely not only on their ability to improve RGC survival rates but also on their protective effects on retinal functional indicators like ERG waveforms and retinal structure. The application of multi-drug combined sustained-release systems, such as PLGA microspheres, greatly promotes the clinical translation and long-term efficacy maintenance of neuroprotective drugs. In the future, combining AI technology with advanced drug delivery systems will accelerate the discovery and optimization of novel neuroprotective agents, thereby providing more effective treatment strategies for glaucoma patients [75,77,78].

### 4.3. Role of Inflammation and Immune Regulation in Neuroprotection

Glaucoma, as a complex neurodegenerative disease, involves multidimensional regulation of immune-inflammatory responses beyond just IOP elevation. Extensive animal model studies show that inflammatory factors like interleukin-6 (IL-6) and tumor necrosis factor-alpha (TNF-α) undergo dynamic changes during glaucoma pathology and are closely related to RGC injury. Taking the CD3ζ-mediated T-cell receptor (TCR) signaling pathway as an example, research found it regulates RGC apoptosis and necrosis in a mouse optic nerve crush model. Knockdown of CD3ζ significantly reduced levels of pro-inflammatory factors like IL-1β and TNF-α while increasing the anti-inflammatory cytokine IL-10, creating a retinal microenvironment conducive to neuroprotection, thereby effectively promoting RGC survival [79]. This suggests the expression of inflammatory factors in glaucoma exhibits complex spatiotemporal dynamics, and immune regulation imbalance is a significant driver of RGC degeneration.

Additionally, the complement system, as a key part of innate immunity, contributes to glaucomatous neurodegeneration through activation-induced inflammatory responses. In the DBA/2J glaucoma mouse model, deficiency of the C3a receptor 1 (C3ar1) significantly reduced the risk of optic nerve degeneration, although it did not affect IOP elevation. It alleviated neuroinflammation by regulating microglial immune response gene expression and its interaction with the anti-inflammatory IL-10 signaling pathway, suggesting complement-mediated inflammation is a key link in glaucomatous neural injury [80].

In the context of oxidative stress and inflammation interaction, NOX2-driven ROS generation regulates endothelial cell release of endothelin-1 (ET-1), activating the ERK1/2 signaling pathway, inducing microglial polarization towards the pro-inflammatory M1 phenotype, thereby triggering a neuroinflammatory storm, leading to blood–retinal barrier (BRB) disruption and RGC loss. Genetic deletion of NOX2 or application of the specific inhibitor gp91ds-tat effectively alleviated these pathological changes, indicating the NOX2/ET-1/ERK1/2 axis is a potential target for regulating the inflammatory microenvironment and neuroprotection [81]. These studies fully demonstrate that the interaction between inflammatory factors and immune cells is a significant component of glaucoma pathology.

Regarding immune regulation strategies, immunomodulators like anti-HMGB1 antibodies have been proven effective in reducing neuroinflammation and protecting RGCs. HMGB1, as a DAMP, is released during retinal I/R injury, activating immune cells and triggering inflammatory responses. Inhibiting HMGB1 signaling can reduce microglial activation, inhibit pro-inflammatory factor release, thereby slowing RGC apoptosis. Similarly, overexpression of the transcription factor ATF3 can inhibit M1 polarization of microglia, reduce the release of inflammatory factors, promote RGC survival, showing good neuroprotective potential [82]. Furthermore, immune response-modulating molecules like PF4 also demonstrate the ability to protect RGCs by inhibiting microglial inflammatory responses and activating the CaMKII/CREB/BDNF neuroprotective pathway, indicating immune regulation is not limited to reducing inflammation but can also activate endogenous neuroprotective mechanisms [41].

In summary, the dynamic changes in inflammatory factors like IL-6 and TNF-α and their mediated immune responses in glanimal models are key links in RGC injury and protection. By targeting inflammation and immune signals like CD3ζ, C3aR1, and the NOX2 pathway, and applying immunomodulatory strategies such as anti-HMGB1 antibodies and ATF3 overexpression, neuroinflammation can be effectively reduced, promoting RGC survival and showing good neuroprotective effects. These studies provide a solid theoretical and experimental basis for the multidimensional mechanism analysis and development of novel therapeutic strategies in glaucoma [41,79,80,81,82].

## 5. Application of Glanimal Models in Fibrosis and Surgical Complication Research

Fibrosis, particularly at the trabecular meshwork and following filtration surgery, is a major pathological process that contributes to aqueous outflow resistance and surgical failure. Animal models of glaucoma filtration surgery (GFS) and TGF-β-driven fibrosis provide essential systems to understand the molecular basis of scarring and to test anti-fibrotic agents. This section discusses the mechanisms of ocular fibrosis and the development of anti-scarring therapies validated in these models.

### 5.1. Fibrosis Mechanisms and Their Manifestations in Animal Models

The fibrotic process holds a significant position in the pathogenesis of glaucoma, especially in the pathological remodeling of ocular tissues. The transforming growth factor-beta (TGF-β) family is a core factor regulating ocular fibrosis, particularly the TGF-β1 and TGF-β2 isoforms, which exhibit significant pro-fibrotic activity in animal models. TGF-β activates the Smad signaling pathway, inducing the transformation of connective tissue cells into myofibroblasts, promoting the deposition of extracellular matrix (ECM) components like collagen and fibronectin, leading to tissue hardening and dysfunction. For example, in a rat glucocorticoid-induced glaucoma model, TGF-β1 expression was significantly elevated, accompanying the progression of retinal fibrosis. Using TGF-β1 antagonists like Oxymatrine (OMT) effectively inhibited the fibrotic process, reduced retinal protein content, restored antioxidant enzyme activity, and alleviated inflammation, showing potential in protecting retinal function [83]. Additionally, activation of the transcription factors Smad2/3 is a key step in TGF-β-mediated fibrosis; regulating this signaling pathway in animal experiments significantly inhibited excessive proliferation of fibrous tissue [84].

In animal models of GFS, fibrosis manifests as abnormal proliferation of connective tissue at the surgical site, leading to blockage of the filtration channel and subsequent postoperative IOP elevation, becoming a primary cause of surgical failure. Rabbits and rats are commonly used GFS animal models due to their suitable eye structure for surgical manipulation and ability to simulate human fibrotic responses. Studies show that increased local TGF-β expression after surgery promotes the transformation of fibroblasts into myofibroblasts, causing collagen deposition and scar formation at the surgical site, directly affecting the survival and function of the filtering bleb [85]. Anti-fibrotic drugs like Mitomycin C (MMC) can effectively reduce scar formation but carry risks of tissue toxicity and complications. Thus, novel therapeutic strategies like using polyunsaturated fatty acid DHA, specific signaling pathway inhibitor FDI-6, and small molecule drug DiOHF have been developed for GFS animal models to alleviate fibrotic responses [84,86,87]. These studies not only validate the core role of the TGF-β signaling pathway in postoperative fibrosis but also suggest that precise regulation of its related pathways may improve the success rate and safety of GFS.

In summary, the high expression of the TGF-β family and its mediated signaling pathways are the main drivers of ocular fibrosis in animal models. The fibrotic process not only exacerbates glaucoma pathology but also significantly affects the efficacy of filtration surgery. Future research needs to continue exploring TGF-β-related molecular mechanisms and combine gene therapy, small molecule drugs, and novel drug delivery systems to explore safer and more effective anti-fibrotic treatment strategies [88,89].

### 5.2. Development and Animal Experimental Validation of Anti-Fibrotic Drugs

Anti-fibrotic drugs play a key role in preventing and controlling scar formation after glaucoma surgery. In recent years, research on various anti-fibrotic drugs like polyenoic acids, docosahexaenoic acid (DHA), and anti-TGF-β antibodies in animal models has deepened, providing new ideas and strategies for further optimizing the long-term outcomes of glaucoma surgery. Studies find that scar tissue in the postoperative filtration channel is mainly caused by abnormal proliferation of fibroblasts and excessive ECM deposition, a process significantly regulated by the TGF-β signaling pathway. Targeting this mechanism, anti-TGF-β antibodies can effectively block TGF-β-induced expression of ECM proteins like fibronectin and type I collagen, thereby inhibiting the fibrosis process of the filtering bleb and maintaining the patency and function of the filtration channel [90].

Besides antibody-based drugs, natural products and their derivatives also show good potential in the anti-fibrosis field. For example, Astragaloside IV significantly lowers IOP in a mouse model and exhibits dual anti-fibrotic and IOP-lowering effects by inhibiting TGF-β2-induced ECM deposition and endoplasmic reticulum stress [90]. Additionally, paclitaxel-like drugs such as sinomenine (SIN) have been confirmed to block the transdifferentiation of fibroblasts into myofibroblasts by inhibiting the NF-κB and PI3K/Akt/mTORC1 signaling pathways, reducing postoperative filtering bleb scar formation [91]. This multi-target, multi-pathway regulatory mechanism provides a theoretical basis for anti-fibrotic therapy.

In terms of innovation in drug delivery systems, researchers have developed various novel carriers to achieve sustained release and targeted action of drugs. For instance, Chitosan-Artesunate nanoparticles (CS@ART NPs), by encapsulating artesunate, improve its solubility and stability, enabling effective inhibition of collagen deposition and fibroblast activity in the rabbit filtering bleb with a single postoperative intracameral injection, while significantly suppressing inflammatory responses, enhancing anti-fibrotic efficacy [92]. Similarly, γ-cyclodextrin cross-linked hydrogel carriers successfully prolonged the release time of the hydrophobic anti-fibrotic drug josamycin, showing good biocompatibility and drug persistence, suitable for local drug delivery after glaucoma surgery [93].

Furthermore, polycaprolactone (PCL) nanofibers were used as drug carriers loaded with the anti-fibrotic drug Pirfenidone, significantly inhibiting fibroblast collagen synthesis in vitro, showing potential for inhibiting scar formation, but they induced strong inflammatory reactions in vivo, suggesting the need for further optimization of carrier materials [94]. Micro-implants like silicone-based microstents provide a new platform for local drug delivery and mechanical support, potentially combining with anti-fibrotic drugs to achieve long-term maintenance of filtration channel function [95].

Overall, current anti-fibrotic drug development not only focuses on the anti-scarring efficacy of the drugs themselves but also emphasizes innovation in drug delivery systems to improve efficacy and safety. Animal experimental validation shows that various anti-fibrotic drugs can effectively inhibit the fibrosis process of the filtering bleb, reduce fibroblast proliferation and excessive ECM deposition, significantly prolonging surgical success rates. Simultaneously, safety evaluations indicate that at reasonable doses and delivery methods, these drugs have low toxicity and side effects on ocular tissues, possessing good clinical translation potential [96]. Future research should further integrate multi-cellular type fibrotic processes, optimize drug combinations and delivery systems, and promote the application of anti-fibrotic treatment strategies in glaucoma surgery.

### 5.3. Establishment and Optimization of Filtration Surgery Animal Models

The glaucoma filtration surgery (GFS) animal model is a key tool for studying the mechanisms of glaucoma surgery and anti-scarring treatment strategies. Mice, rats, and rabbits are currently commonly used animal models in GFS research, each with unique advantages and disadvantages. Mice and rats, as small rodents, are often used to study fibrotic responses post-filtration surgery and screen novel anti-fibrotic drugs due to their rapid reproduction, low cost, and mature genetic manipulation techniques. Their eyes are small, making surgical manipulation more challenging, but they are suitable for large-scale drug screening and molecular mechanism research [85]. In contrast, rabbits have larger eye sizes and structures closer to humans, making them suitable for developing surgical techniques and testing medical devices. The rabbit model is more convenient for GFS manipulation and observation and can better simulate the human postoperative fibrotic process [97,98]. However, rabbit models are more expensive and require higher maintenance, and their wound healing rate is faster than humans, which may affect long-term surgical outcome evaluation.

**Figure 4:**
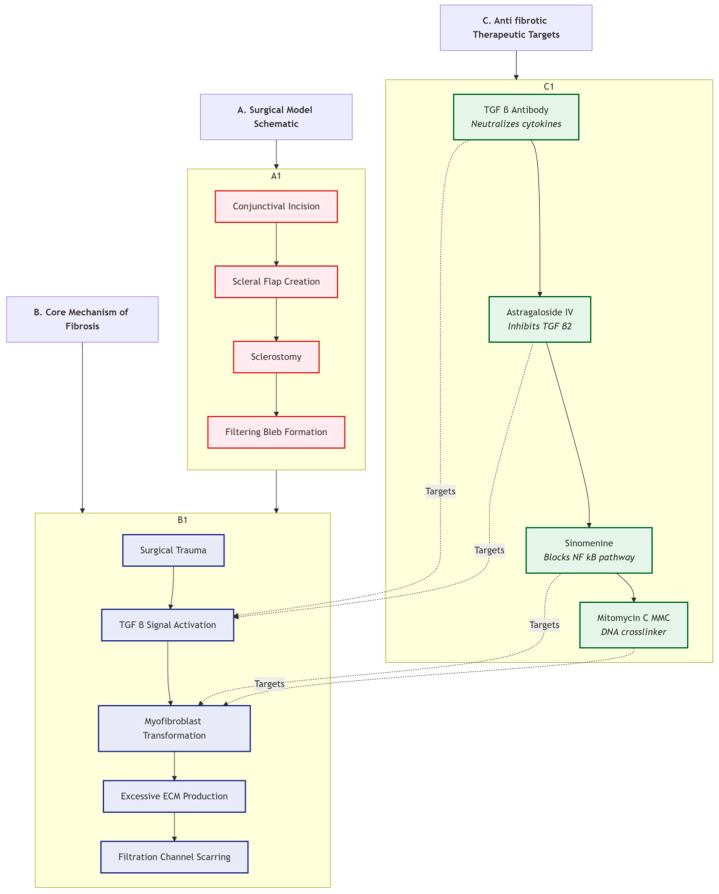
Glaucoma Filtration Surgery Animal Model and Anti-fibrotic Treatment Strategies.

Figure 4 illustrates the glanimal filtration surgery model and anti-fibrotic strategies. Part A shows a schematic diagram of the surgical model, depicting key steps of filtration surgery; Part B illustrates the core mechanisms of fibrosis, showing the entire process from surgical trauma to TGF-β signaling activation, ultimately leading to excessive ECM production and filtration channel scarring; Part C presents anti-fibrotic therapeutic targets, including the mechanisms of action of drugs like TGF-β antibody, Astragaloside IV, Sinomenine, and MMC.

In constructing GFS animal models, standardizing surgical procedures and pre-inducing a glaucomatous state are key factors for improving the consistency and clinical relevance of research results. Currently, most studies perform filtration surgery directly on normal eyes, but this overlooks the impact of the glaucomatous pathological state on surgical healing and scar formation. Pre-inducing a glaucomatous state, such as through repeated high IOP treatment or laser-induced damage, places the animal’s eye tissue in a pathological environment similar to clinical patients, helping to more realistically reflect postoperative fibrosis and inflammatory responses [85]. Additionally, standardizing operations such as the dosage and administration methods (e.g., sponge application vs. subconjunctival injection) of anti-fibrotic drugs during and after surgery significantly affects the stability and data reliability of the filtration surgery model. For instance, in rabbit filtration surgery, subconjunctival injection of MMC more effectively lowers IOP, reduces angiogenesis and fibrosis, and alleviates MMC-related toxic side effects compared to sponge application, suggesting that optimizing the administration method is crucial for model construction [97].

Future research should focus on establishing filtration surgery animal models that more closely resemble the clinical pathological state, combining pre-treated glaucomatous conditions and strict surgical standardization procedures to enhance the clinical translational value of animal models. Simultaneously, the comprehensive application of multi-species models will help fully assess the safety and efficacy of novel anti-scarring drugs and treatment strategies, providing a solid experimental foundation for optimizing clinical glaucoma filtration surgery [85,99].

## 6. Application of Advanced Imaging Technologies and Functional Assessment in Glanimal Models

The objective and longitudinal assessment of disease progression and treatment efficacy is critical in glaucoma research. Technological advances have provided powerful tools for non-invasive, in vivo monitoring of structural and functional changes in animal models. This section highlights key technologies such as Diffusion Tensor Imaging, electroretinography, and optical coherence tomography, and emphasizes the value of multimodal integration for comprehensive evaluation.

**Figure 5:**
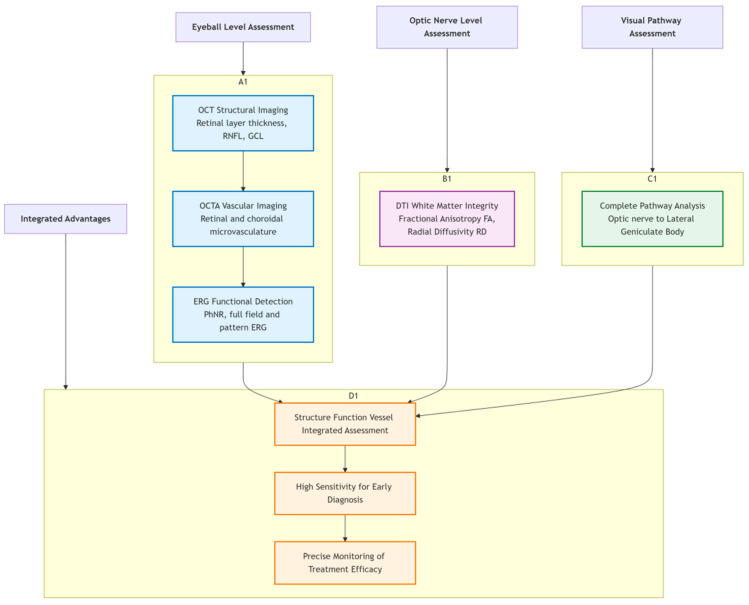
Application of Advanced Imaging and Functional Assessment Technologies in Glaucoma Models.

Figure 5 summarizes the multimodal assessment techniques used in glanimal models. Assessment at the eyeball level includes OCT structural imaging, OCTA vascular imaging, and ERG functional detection; assessment at the optic nerve level evaluates white matter integrity via DTI; visual pathway assessment covers the complete pathway from the optic nerve to the lateral geniculate body. The bottom summarizes the advantages of technology integration: achieving integrated structure-function-vessel assessment, with high sensitivity for early diagnosis and precise monitoring of treatment effects.

### 6.1. Diffusion Tensor Imaging (DTI) Technology

Diffusion Tensor Imaging (DTI) is an advanced magnetic resonance imaging (MRI)-based technique that reflects the microstructural integrity of tissues by measuring the diffusion characteristics of water molecules within biological tissues. In glaucoma research, DTI is widely used to assess microstructural changes in the optic nerve and visual pathways, particularly the integrity of optic nerve fibers. Glaucoma not only causes the death of RGCs and their axons but also leads to degeneration of the optic nerve and subsequent visual pathway structures, changes that can be detected and quantified non-invasively through DTI parameters [100]. DTI can cover the entire visual system, including the optic nerve, optic chiasm, optic tract, lateral geniculate body, and optic radiations, revealing the characteristics of nerve fiber damage and degeneration caused by glaucoma by analyzing metrics like fractional anisotropy (FA) [100,101].

In different glanimal models, DTI parameters exhibit specific patterns of change. For example, a study of five glanimal models (including chronic IOP elevation models induced by microbead injection or laser photocoagulation, and genetic models like DBA/2J mice, LTBP2 mutant cats, and TBK1 transgenic mice) showed that chronic IOP elevation is typically accompanied by a significant decrease in FA and a significant increase in radial diffusivity (RD) in the optic nerve and optic tract, reflecting axonal and myelin structural damage [101]. DBA/2J mice also showed a decrease in axial diffusivity (AD), suggesting more severe axonal structural damage, indicating heterogeneity in pathological mechanisms among different models [101]. In contrast, TBK1 transgenic mice showed no significant DTI parameter changes, possibly due to their insignificant IOP changes [101].

DTI metrics are closely related to glaucoma pathological progression and functional impairment. Decreased FA and increased RD typically correspond to impaired microstructural integrity of the optic nerve, reflecting axonal degeneration and myelin injury; while changes in AD may indicate different stages of axonal injury [101,102]. Furthermore, changes in DTI parameters correlate with clinical ophthalmic indicators like RNFL thickness and visual field defect severity, indicating DTI can serve as an effective biomarker for assessing optic neuropathy and predicting visual function loss [103,104]. In a canine angle-closure glaucoma model, DTI also showed decreased FA and increased RD in the optic nerve, chiasm, tract, and lateral geniculate body, suggesting good applicability and reproducibility of DTI technology across different animal models [105].

Notably, the continuous advancement of DTI technology and its combination with artificial intelligence provide new perspectives and higher sensitivity for glaucoma diagnosis and research. DTI can not only reveal microstructural changes in the optic nerve but also reflect broader brain structural abnormalities, supporting the view of glaucoma as a whole-brain neurodegenerative disease [100,106]. In summary, DTI technology, as a non-invasive, high-resolution imaging method, provides an important tool and theoretical basis for in-depth understanding of the multidimensional pathological mechanisms of glaucoma and the development of early diagnosis and treatment strategies.

### 6.2. Electrophysiological Detection (Erg) and Retinal Function Assessment

Electrophysiological detection techniques, particularly electroretinography (ERG), hold significant application value in glanimal model research. ERG can objectively assess retinal function, especially the functional status of RGCs, providing a sensitive means for detecting early ischemic injury. Multiple studies indicate that changes in ERG parameters can reflect the progressive impairment of retinal function, thereby helping to reveal the dynamic process of neurodegenerative changes during glaucoma pathology.

Firstly, the value of ERG in detecting retinal neural function and early ischemic injury is highly significant. For example, in a chronic glaucoma model induced by a single injection of fibronectin-loaded PLGA microspheres, the recorded retinal functional impairment by ERG showed a progressively worsening trend. ERG detected functional changes more sensitively than optical coherence tomography (OCT) detected structural damage, especially in the presence of significant astrocyte gliosis, where ERG could reveal dysfunction early [107]. Additionally, in microbead-induced IOP elevation models, the reduction in the photopic negative response (PhNR) amplitude of ERG is closely related to RGC functional impairment, reflecting damage to the retinal nerve fiber layer [108,109]. ERG has also been used to evaluate functional changes in retinal ischemia models and optic nerve crush models. For instance, in an NMDA-induced retinal injury monkey model, significant alterations in ERG waveforms synchronized with RGC loss, demonstrating the applicability of this technique in non-human primate models [110]. Notably, ERG can not only reflect overall retinal function but also distinguish the functional status of ON and OFF retinal pathways through specific stimulus parameters, which is important for understanding the susceptibility of different RGC subtypes in glaucoma [111].

Secondly, combining ERG exclusion criteria to optimize the screening of animal models helps improve the accuracy and reproducibility of experimental data. Due to the fluctuating nature of IOP in glaucoma models, relying solely on IOP thresholds (e.g., >60 mmHg) as exclusion criteria may not accurately reflect whether retinal ischemic damage has occurred [112]. Studies show that a significant reduction in the b-wave amplitude of ERG can serve as a sensitive indicator for detecting ischemic damage. Applying this ERG standard to exclude animals allows for more precise selection of model animals without ischemic injury, thereby avoiding misclassification of samples and improving the reliability of experimental results [112]. Furthermore, multi-parameter analysis of ERG, combined with waveform texture features and multivariate statistical models, can more comprehensively predict glaucoma severity, providing finer tools for model evaluation and treatment effect monitoring [113,114]. In long-term follow-up of chronic glaucoma models, ERG changes significantly correlate with RNFL thickness and visual function decline, providing multi-dimensional functional indicators for model evaluation [115,116]. Additionally, the application of ERG extends to evaluating the effects of neuroprotective therapies, such as nattokinase, PACAP eye drops, and neurotrophic factors delivered via nanoparticle carriers, all of which have demonstrated retinal protective effects validated by ERG functional recovery [77,109,117].

In summary, ERG, as a gold standard technique for retinal functional assessment in glanimal models, can sensitively detect early retinal ischemic and neural functional injury. Establishing scientific exclusion criteria based on ERG optimizes animal model screening, significantly improving the accuracy and reproducibility of experimental data. In the future, combining ERG multi-parameter analysis with structural imaging technologies will further deepen the understanding of glaucoma pathological mechanisms and promote the development and evaluation of novel therapeutic strategies.

### 6.3. In Vivo Optical Coherence Tomography (Oct) and Other Imaging Techniques

In vivo optical coherence tomography (OCT), as a non-invasive, high-resolution imaging technique, has become a core tool in glaucoma research and clinical diagnosis. It can dynamically monitor changes in retinal layer thickness and the nerve fiber layer, providing important basis for early diagnosis and disease progression assessment of glaucoma. In recent years, with continuous advancements in OCT technology, including the development of various modes like adaptive optics OCT (AO-OCT), spectral-domain OCT (SD-OCT), and swept-source OCT (SS-OCT), observation of microstructures in the retina and optic nerve head has become more precise and in-depth.

OCT exhibits high sensitivity in dynamically monitoring changes in the retinal nerve fiber layer (RNFL) and ganglion cell layer (GCL) thickness. For example, using SD-OCT, three-dimensional imaging of the optic nerve head and RNFL can be achieved, not only quantifying RNFL thickness but also observing morphological changes in the optic disk, such as cupping and excavation, which are important indicators of glaucoma progression [118]. Furthermore, the introduction of AO-OCT technology enables researchers to directly observe the morphological structure of RGC bodies and nerve fiber bundles at a cellular level resolution in the living eye, revealing micro-pathological changes in glaucoma and other neurodegenerative diseases [119]. This multi-parameter imaging provides multidimensional information like reflectivity, delay, and angiography, enhancing the capability for early glaucoma detection.

On the other hand, the application of OCT has extended to the assessment of glaucoma-related structural abnormalities, such as the morphology and biomechanical properties of the lamina cribrosa (LC). Studies show that changes in LC thickness reduction, local defects, and posterior displacement in glaucoma patients can be detected by OCT. These parameters not only aid in disease diagnosis but can also predict disease progression [120,121]. Simultaneously, OCT combined with angiography (OCTA) can non-invasively assess changes in the retinal and optic nerve head microvascular system, finding insufficient perfusion in retinal and choroidal vessels in early glaucoma patients, providing important evidence for the vascular pathological mechanism research of glaucoma [118,122].

With the integration of AI technology, deep learning algorithms based on OCT images have demonstrated excellent performance in the automatic diagnosis and progression detection of glaucoma. These algorithms can effectively process high-dimensional 3D OCT data, achieving precise identification and quantification of RNFL and optic disk structures, greatly improving the efficiency of clinical screening and follow-up [123,124]. Furthermore, combining multimodal OCT with retinal functional tests, such as visual field testing and electrophysiological assessment, helps establish structure-function correlation models, further improving the comprehensive evaluation system for glaucoma [125].

In summary, OCT and its derivative technologies play an irreplaceable role in the dynamic monitoring of glaucoma. Through real-time, high-resolution imaging, they allow detailed observation of microstructural changes in the RNFL and optic nerve head, capturing early pathological signals of the disease in a timely manner. Simultaneously, combined with vascular imaging and AI analysis, they provide multi-dimensional, multi-level comprehensive assessment means, promoting the development of precision diagnosis and treatment in glaucoma.

### 6.4. Comprehensive Assessment Methods for Glaucoma Progression Using Multimodal Imaging

Facing the complex pathological mechanisms and multidimensional clinical manifestations of glaucoma, a single imaging technique often struggles to comprehensively reflect the disease state. The combination of multimodal imaging technologies provides new ideas and methods for the comprehensive assessment of glaucoma. By integrating structural OCT imaging, OCT angiography (OCTA), retinal function measurements, and other auxiliary imaging methods, multi-angle, multi-level monitoring of glaucoma progression can be achieved.

Firstly, structural OCT imaging provides quantitative information on RNFL thickness, GCL, and optic disk morphology, forming the basis for judging glaucomatous neurodegeneration [118,126]. Secondly, OCTA can non-invasively observe changes in the retinal and choroidal microvascular networks, detecting vascular injuries like insufficient blood flow perfusion and reduced capillary density, providing important physiological evidence for vascular glaucoma and its progression [122,127]. Combining the two allows simultaneous assessment of neural structural integrity and vascular supply status, revealing the neurovascular compound pathological process in glaucoma.

Furthermore, combining functional tests, such as visual evoked potentials (VEP), pattern ERG (PERG), and visual field testing, can reflect optic nerve conduction function and visual field defects, further supplementing the shortcomings of structural imaging [128,129]. This multimodal combination of structure and function helps establish predictive models for glaucoma progression, enabling early intervention and individualized management.

In recent years, advanced multimodal imaging also includes OCT combined with adaptive optics (AO) and scanning laser ophthalmoscopy (SLO), enabling high-resolution imaging at the cellular level of the retina, effectively resolving RGC morphology and vascular microstructure [129,130]. The fusion of these technologies allows clinicians to accurately identify early glaucomatous lesions, monitor treatment effects, and explore new biomarkers.

Additionally, the application of multimodal imaging technologies is increasingly widespread in animal model research. For example, using OCT combined with immunofluorescence staining and histological methods deeply analyzes microstructural changes in the glaucomatous optic nerve head and retina, providing important pathological basis for clinical practice [101,131]. Simultaneously, combining with MRI techniques like diffusion tensor imaging (DTI) provides a systemic assessment of glaucoma from the perspective of nerve fiber tract integrity [101,106].

In summary, the multimodal imaging assessment method for glaucoma, through the multi-level fusion of structure, vessels, and function, breaks through the limitations of single imaging techniques. Such comprehensive assessment not only enhances the sensitivity and specificity of early disease diagnosis but also supports accurate judgment of disease progression through dynamic monitoring, promoting the development of individualized treatment plans and efficacy evaluation for glaucoma, marking the entry of glaucoma clinical management into a new multidimensional and precise stage.

## 7. Conclusions

This review systematically summarizes the establishment, application, and future trends of diverse glanimal models in glaucoma research. The key strengths and contributions of this work include: (1) A comprehensive classification and comparative analysis of major glaucoma animal models, highlighting their specific applications and limitations; (2) An in-depth discussion of the multidimensional pathogenesis of glaucoma elucidated through these models, encompassing mechanical, immune, excitotoxic, and ischemic mechanisms; (3) A detailed overview of the critical role of animal models in screening neuroprotective agents, evaluating anti-fibrotic strategies, and validating advanced imaging and functional assessment technologies; (4) The proposition of future research directions focused on model standardization, multi-factor simulation, and the integration of novel drug delivery systems and immunomodulatory strategies.

**Figure 6:**
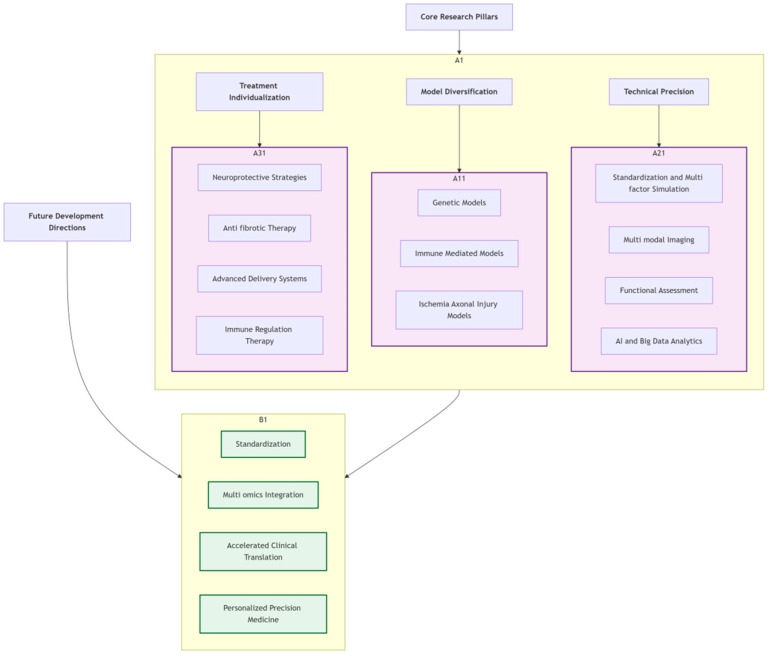
Overall Strategy and Future Development Directions for Glaucoma Research.

Figure 6 summarizes the overall strategy and future directions for glaucoma research. Achieving comprehensive disease coverage through model diversification, enhancing research depth through technological precision, and promoting clinical translation through treatment individualization. The future should focus on standardization construction, multi-omics integration, accelerating clinical translation, and achieving personalized precision medicine, ultimately providing more effective prevention and treatment solutions for glaucoma patients.

Glaucoma, as a complex optic neuropathy under the action of multiple factors, involves various aspects of pathology including genetics, mechanical pressure, immune responses, and metabolic abnormalities. Existing animal models, by simulating these different pathological pathways, provide us with a relatively comprehensive disease reproduction platform, enabling researchers to deeply explore the specific mechanisms of RGC injury at physiological and pathological levels. These models not only cover genetic diversity but also integrate factors like mechanical IOP elevation, immune-mediated neuroinflammation, and metabolic disorders, demonstrating the multidimensional characteristics of glaucoma and greatly enriching our understanding of disease progression.

From an expert perspective, diversified animal models have brought significant breakthroughs to glaucoma research. On one hand, these models provide a solid experimental foundation for screening and evaluating neuroprotective and anti-fibrotic drugs, making the drug development process more precise and efficient; on the other hand, model diversity also promotes systematic research on the interactions between different pathological mechanisms, helping to clarify key nodes in the complex pathological network. However, different models have certain differences in their focus and pathological manifestations when simulating disease features, posing challenges for interpreting experimental results and clinical translation. Therefore, how to balance and integrate the advantages of these models, avoiding over-reliance on a single model, is an urgent problem to be solved in future research.

Combining advanced imaging technologies and functional detection methods, such as OCT, multi-photon microscopy, and electrophysiological detection, has greatly enhanced the ability to dynamically monitor pathological progression in animal models. These technologies not only enable precise quantification of RGC injury, optic nerve fiber changes, and visual dysfunction but also provide objective and repeatable evaluation indicators for treatment effects. Through the integration of multimodal detection technologies, researchers can more comprehensively understand the micro-changes during disease development, promoting close connections between basic research and clinical applications.

Looking forward, the development of glanimal models should pay more attention to standardization and multi-factor joint simulation. Standardization not only helps improve the comparability and reproducibility of experimental results but can also accelerate cross-laboratory collaboration and data sharing. Multi-factor joint simulation is closer to clinical reality, capable of reflecting the complex and variable pathological state of glaucoma, thereby enhancing the clinical relevance and translational potential of models. Furthermore, the successful application of novel drug carrier technologies and immune regulation strategies in animal models opens new directions for clinical treatment. Carrier technologies like nanoparticles and sustained-release systems improve drug targeting and bioavailability, while immune regulation provides new ideas for slowing inflammation-mediated optic nerve injury. The integration of these innovative means is expected to break through the bottlenecks of traditional treatment and achieve individualized precision therapy for glaucoma.

In summary, the diversification of glanimal models and the advancement of technological means have greatly promoted in-depth analysis of disease mechanisms and optimization of treatment strategies. Future research should focus on the standardized construction of models and the integrated simulation of multiple pathological mechanisms, while actively introducing advanced imaging and functional assessment technologies to strengthen the bridge between basic research and clinical application. By comprehensively utilizing novel drug carriers and immune regulation strategies, the therapeutic prospects for glaucoma will be broader, bringing more effective protection and vision recovery possibilities to patients. As senior researchers in the medical field, we should continuously promote the development of this field, facilitate the organic combination of theoretical innovation and clinical practice, and ultimately achieve a qualitative leap in the level of glaucoma prevention and treatment.

## Figures and Tables

**Table 1:** Comparison of Establishment Methods and Applications of Major Glanimal Models.

Model Type	Representative Methods	Simulated Pathological Features	Advantages	Limitations
**Genetic Models**	DBA/2J mouse, LTBP2 mutant cat	Angle dysgenesis, Chronic IOP elevation	Spontaneous onset, Close to human genetic background	Uncontrollable course, High cost, Long cycle
**IOP Elevation Models**	Microbead injection, Laser photocoagulation, Steroid induction	Aqueous outflow obstruction, Acute/Chronic ocular hypertension	Controllable IOP, Relatively simple operation	Potential surgical trauma, Non-specific injury
**Immune-Mediated Models**	Autoantigen immunization, NMDA injection	Neuroinflammation, Excitotoxicity	Reveals non-IOP mechanisms, Suitable for immune research	Relatively singular pathology, Clinical relevance needs validation
**Ischemia/Axonal Injury Models**	Ischemia–reperfusion, Optic nerve crush	Ischemia/hypoxia, Acute axonal injury	Rapid induction, Suitable for neuroprotection research	Acute models, Differ from chronic progression

## Data Availability

No datasets were generated or analyzed during the current study.

## Abbreviations

AD	Axial Diffusivity
AI	Artificial Intelligence
AO-OCT	Adaptive Optics Optical Coherence Tomography
BDNF	Brain-Derived Neurotrophic Factor
BRB	Blood–Retinal Barrier
CaMKII	Calcium/Calmodulin-Dependent Protein Kinase II
CNTF	Ciliary Neurotrophic Factor
CREB	cAMP Response Element-Binding Protein
CRISPR	Clustered Regularly Interspaced Short Palindromic Repeats
DAMP	Damage-Associated Molecular Pattern
DHA	Docosahexaenoic Acid
DTI	Diffusion Tensor Imaging
ECM	Extracellular Matrix
ERG	Electroretinography
ET-1	Endothelin-1
FA	Fractional Anisotropy
FABP5	Fatty Acid-Binding Protein 5
GCL	Ganglion Cell Layer
GDNF	Glial Cell Line-Derived Neurotrophic Factor
GFS	Glaucoma Filtration Surgery
HMGB1	High Mobility Group Box 1
IOP	Intraocular Pressure
I/R	Ischemia–Reperfusion
IL	Interleukin
iPSC	Induced Pluripotent Stem Cell
KYNA	Kynurenic Acid
KYN	Kynurenine
LC	Lamina Cribrosa
MAPK	Mitogen-Activated Protein Kinase
MMC	Mitomycin C
MRI	Magnetic Resonance Imaging
NMDA	N-methyl-D-aspartate
NOX	NADPH Oxidase
NTG	Normal Tension Glaucoma
OCT	Optical Coherence Tomography
OCTA	Optical Coherence Tomography Angiography
ONA	Optic Nerve Antigen
ONC	Optic Nerve Crush
PCL	Polycaprolactone
PERG	Pattern Electroretinography
PhNR	Photopic Negative Response
PLGA	Poly(lactic-co-glycolic acid)
POAG	Primary open-angle glaucoma
RAAS	Renin–Angiotensin–Aldosterone System
RD	Radial Diffusivity
RGC	Retinal Ganglion Cell
RIP1	Receptor-Interacting Protein Kinase 1
RNFL	Retinal Nerve Fiber Layer
ROS	Reactive Oxygen Species
SD-OCT	Spectral-Domain Optical Coherence Tomography
SLT	Selective Laser Trabeculoplasty
SLO	Scanning Laser Ophthalmoscopy
SPP1	Secreted Phosphoprotein 1 (Osteopontin)
SS-OCT	Swept-Source Optical Coherence Tomography
SYN1	Synapsin I
TCR	T-cell Receptor
TGF-β	Transforming Growth Factor-Beta
TNF-α	Tumor Necrosis Factor-Alpha
TRP	Tryptophan
VEP	Visual Evoked Potential
